# Human induced pluripotent stem cell for modeling cardiovascular diseases

**DOI:** 10.1186/2050-490X-2-4

**Published:** 2014-02-05

**Authors:** Ping Liang, Jie Du

**Affiliations:** Beijing Anzhen Hospital, Capital Medical University, Beijing, China; The key laboratory of Remodeling-related Cardiovascular Disease, Ministry of Education, Capital Medical University, Beijing, China; Beijing Institute of Heart Lung & Blood Vessel Disease, Beijing, China; Department of Medicine, Division of Cardiology, Stanford University, Stanford, CA USA; Institute for Stem Cell Biology and Regenerative Medicine, Stanford University, Stanford, CA USA; Cardiovascular Institute, Stanford University, Stanford, CA USA; Lorry I. Lokey Stem Cell Research Building, Rm G1105, 265 Campus Drive, Stanford, CA 94305 USA

**Keywords:** Stem cells, Induced pluripotent stem cells, Cardiovascular disease, Disease modeling

## Abstract

The invention of the induced pluripotent stem cell (iPSC) technology allows patient-specific, mature somatic cells to be converted into an unlimited supply of pluripotent stem cells (PSCs). These iPSCs can then in turn be differentiated into any cell type including neurons, cardiac cells, pancreatic cells, liver cells, blood cells or enterocytes. Although cardiovascular disease (CVD) is a leading cause of death in the world, the limited cell derivation and cell number in cardiac tissue makes it difficult to study the CVDs using the existing cardiac cell model. By differentiating the patient-specific iPSCs into cardiomyocytes, scientists can generate iPSC-based 'disease in a dish’ models and use them to better understand disease mechanism. Here we review the current progress in using iPSC-derived cardiomyocytes to model human CVDs.

## Pluripotent stem cells (PSCs) for translational medicine

Recently, human embryonic stem cells (hESCs), used as pluripotent cells, were widely considered a useful resource in translational medicine. In 1998, hESCs were isolated from inner cells of blastocysts and characterized for their pluripotency and self-renewal [1]. In contrast to adult stem cells, hESCs can be differentiated into any type of functional cells. The applications of hESCs in translational medicine, however, are controversial, and it is difficult to suppress immunologic rejection in hESC-based therapies [2].

## IPSC: a game changer

Two groups (Yamanaka group in Kyoto University and Thomson group in University of Wisconsin) separately reported that somatic cells can be successfully reprogrammed into iPSCs, marking a major landmark in stem cell research [3, 4]. In each study, four transcription factors were used for induction. Yamanaka’s group selected Oct3/4, Sox2, Klf, and c-Myc [3], while. Thomson’s group used Oct3/4, Sox2, Nanog, and Lin28 [4]. This allowed researches to reprogram mature somatic cells harvested from patients and generate an unlimited supply of PSCs which in turn could be differentiated into various cell types needed such as neurons, cardiac cells, pancreatic cells, liver cells, blood cells or enterocytes for disease modeling, drug screening and cell therapy [5–10]. iPS cells have a couple of key advantages: they avoid the ethical concerns that have plagued the embryonic stem cell field, and they are patient-specific, thus providing a powerful tool for translational medicine.

## iPSC for cardiovascular diseases

A straightforward application of iPSCs is to establish patient-specific genetic disease models *in vitro*. These models are useful for understanding mechanism of physiology and pathology of disease, validating therapeutic targets, and drug screening/discovery. CVD, more commonly known as heart disease, encompasses all heart and blood vessel disease. CVD is the world’s leading cause of death; more people die annually from CVD than from any other cause, with an estimated 17.3 million people dying in 2008, accounting for 30 % of all global deaths. CVD encompasses a broad range of disorders such as atherosclerosis, ischemic heart disease, acute myocardial infraction, valvular heart disease, heart failure, cardiomyopathies, arrhythmias, hypertension, and congenital heart disease. By differentiating patient-specific iPSCs into patient-specific cardiomyocytes, researchers can generate iPSC-based 'disease in a dish’ models and use them to better understand disease mechanism and develop new therapeutics [11, 12]. Ion channels are pore-forming membrane proteins whose functions allow them operate like biological micro-machines to establish a resting potential, shape action potentials, and control the excitability of neurons, cardiac cells and muscle cells, thus playing an essential role in functional regulation of the cardiovascular system. The outward currents (such as I_ks_ and I_kr_ encoded by KCNQ1 and human ether-á-go-go–related gene (hERG) gene, respectively) contribute to repolarization of cardiac action potentials, which helps bring membrane potentials back to the resting level and terminate a ventricular contraction. The timing of these repolarizing currents (I_ks_ and I_kr_) is critical. Failure to repolarize due to inherited mutations in KCNQ1 or hERG gene, or drug-induced I_ks_ and I_kr_ inhibitions, for example, can result in abnormalities in action potentials, thus causing Long QT syndrome (Type 1 or 2). Moretti et al. [13] were an early example, taking skin biopsies from patients with Long QT Syndrome Type 1 (LQT-1), reprogramming their cells into iPSCs, and then differentiating those iPSCs into cardiac cells. These patient-specific cardiomyocytes recapitulated the clinical presentation of the 'Long QT’ phenotype [13]. Using a similar approach, [14] successfully modeled Long QT Syndrome Type 2 (LQT-2). These studies clearly established iPSC-derived cardiomyocytes as a powerful tool for drug discovery and personalized medicine [15]. To date, iPSC models have been used to model a large number of genetic arrhythmias including LQT syndromes, catecholaminergic polymorphic ventricular tachycardia (CPVT), arrhythmogenic right ventricular cardiomyopathy (ARVD), and Overlap Syndrome [14, 16–24]. A number of genetic cardiomyopathies have also been studied using iPSCs [25]. generated iPSCs from skin cells of patients in a family with inherited dilated cardiomyopathy (DCM) carrying a mutation in the TNNT2 gene, a gene encodes Troponin T type 2 (cardiac) that is the tropomyosin-binding subunit of the troponin complex and mutations in this gene have been associated with dilated cardiomyopathy familial hypertrophic cardiomyopathy as well as restrictive cardiomyopathy [25]. Compared to cardiomyocytes derived from iPSCs of healthy controls in the same family, cardiomyocytes derived from iPSCs of DCM patients exhibited increased heterogeneous myofilament organization, susceptibility to stress, compromised ability to regulate calcium flux, and decreased contractile force. More recently, Lan et al. reported modeling a familiar hypertrophic cardiomyopathy (HCM) [26]. Patient-specific induced pluripotent stem cell cardiomyocytes (iPSC-CMs) from a ten-member family cohort carrying a hereditary HCM missense mutation (Arg663His) in the MYH7 gene were generated. This gene encodes a myosin heavy chain beta (MHC-β) isoform expressed primarily in the heart. Diseased iPSC-CMs recapitulated numerous aspects of the HCM phenotype including cellular enlargement, abnormal Ca^2+^ handling and contractile arrhythmia at the single-cell level. In addition to those early-onset cardiovascular diseases modeled by iPSC-derived cardiomyocytes mentioned above, Kim et al. for the first time demonstrated late-onset disease models using ARVD/C patient-specific iPSCs, in which the patients don’t have clinical phenotype until adulthood [27]. Several groups have reported that iPS cells can also be differentiated into functional endothelial cells (ECs) or vascular smooth muscle cells (SMCs), which can shed new light up on understanding the mechanisms of various vascular diseases such as hypertension, pulmonary atrial hypertension (PAH), coronary heart disease, and diabetic cardiomyopathy. There are, however, fewer vascular diseases being modeled by iPSCs thank cardiac diseases [28–32]. Kinnear et al. generated iPSCs from a patient with aortic and pulmonary stenosis who was suffering from Williams-Beuren syndrome (WBS), a rare genetic neurodevelopmental disorder that can cause cardiovascular disease [33]. Interestingly, WBS patient-specific iPS cell-derived SMCs demonstrate an immature proliferative phenotype with reduced functional and contractile properties compared to healthy controls, thereby recapitulating the human disease phenotype. Moreover, they showed that the long-term treatment (5 days) of the antiproliferative drug rapamycin can rescue the disease phenotype, providing an attractive therapeutic candidate for patients with WBS and vascular stenosis.

## Review and conclusions

A growing number of research groups have employed iPSCs to model CVDs. It remains unclear whether diseased iPSC-derived cardiomyocytes show disease phenotypes *in vitro* and how similar *in vitro* phenotypes are to their clinical equivalents. It is to be noted that iPSC-derived cardiomyocytes are morphologically and electrophysiologically immature compared to human adult counterparts. Several studies have focused on developing methologies to obtain more mature cardiomyocytes derived from iPSCs [34]. iPSC-derived cardiomyocytes are also a mixture of 3 subtypes of cells (ventricular-like, atrial-like, nodal-like). All three differentiation methods including embryoid body formation, endodermal induction and directed differentiation method reflect these shortcomings. Future challenges will focus on how diseased iPSC models can faithfully reflect disease phenotypes. Published iPSC studies only reproduce disease phenotypes and drugs that have already been reported using other approaches such as transgenetic animal models or primary cells. Moreover, most of the published iPSC disease model works are the ones looking into arrhythmic disorders carried ion channel gene mutations. To demonstrate the power of iPSCs, scientists have to gain more mechanistic insights, discover novel drug targets, and investigate more complicated cardiovascular diseases. In conclusion, although iPSC-based cardiovascular disease research is in its infancy, further improvement and standardization of the culturing methods can lead to a more comprehensive understanding of mechanisms in cardiovascular disease.

## References

[1] Thomson JA, Itskovitz-Eldor J (1998). Embryonic stem cell lines derived from human blastocysts. Science.

[2] Swijnenburg RJ, Schrepfer S (2008). Immunosuppressive therapy mitigates immunological rejection of human embryonic stem cell xenografts. Proc Natl Acad Sci U S A.

[3] Takahashi K, Tanabe K (2007). Induction of pluripotent stem cells from adult human fibroblasts by defined factors. Cell.

[4] Yu J, Vodyanik MA (2007). Induced pluripotent stem cell lines derived from human somatic cells. Science.

[5] Chicha L, Feki A (2011). Human pluripotent stem cells differentiated in fully defined medium generate hematopoietic CD34- and CD34+ progenitors with distinct characteristics. PLoS One.

[6] Kobayashi T, Yamaguchi T (2010). Generation of rat pancreas in mouse by interspecific blastocyst injection of pluripotent stem cells. Cell.

[7] Spence JR, Mayhew CN (2010). Directed differentiation of human pluripotent stem cells into intestinal tissue in vitro. Nature.

[8] Dimos JT, Rodolfa KT (2008). Induced pluripotent stem cells generated from patients with ALS can be differentiated into motor neurons. Science.

[9] Song Z, Cai J (2009). Efficient generation of hepatocyte-like cells from human induced pluripotent stem cells. Cell Res.

[10] Zhang D, Jiang W (2009). Highly efficient differentiation of human ES cells and iPS cells into mature pancreatic insulin-producing cells. Cell Res.

[11] Rosenzweig A (2010). Illuminating the potential of pluripotent stem cells. N Engl J Med.

[12] Yoshida Y, Yamanaka S (2010). Recent stem cell advances: induced pluripotent stem cells for disease modeling and stem cell-based regeneration. Circulation.

[13] Moretti A, Bellin M (2010). Patient-specific induced pluripotent stem-cell models for long-QT syndrome. N Engl J Med.

[14] Itzhaki I, Maizels L (2012). Modeling of catecholaminergic polymorphic ventricular tachycardia with patient-specific human-induced pluripotent stem cells. J Am Coll Cardiol.

[15] Itzhaki I, Maizels L (2011). Modelling the long QT syndrome with induced pluripotent stem cells. Nature.

[16] Davis RP, Casini S (2012). Cardiomyocytes derived from pluripotent stem cells recapitulate electrophysiological characteristics of an overlap syndrome of cardiac sodium channel disease. Circulation.

[17] Fatima A, Xu G (2011). In vitro modeling of ryanodine receptor 2 dysfunction using human induced pluripotent stem cells. Cell Physiol Biochem.

[18] Lahti AL, Kujala VJ (2012). Model for long QT syndrome type 2 using human iPS cells demonstrates arrhythmogenic characteristics in cell culture. Dis Model Mech.

[19] Ma D, Wei H (2013). Generation of patient-specific induced pluripotent stem cell-derived cardiomyocytes as a cellular model of arrhythmogenic right ventricular cardiomyopathy. Eur Heart J.

[20] Matsa E, Rajamohan D (2011). Drug evaluation in cardiomyocytes derived from human induced pluripotent stem cells carrying a long QT syndrome type 2 mutation. Eur Heart J.

[21] Novak A, Barad L (2012). Cardiomyocytes generated from CPVTD307H patients are arrhythmogenic in response to beta-adrenergic stimulation. J Cell Mol Med.

[22] Terrenoire C, Wang K (2013). Induced pluripotent stem cells used to reveal drug actions in a long QT syndrome family with complex genetics. J Gen Physiol.

[23] Yazawa M, Hsueh B (2011). Using induced pluripotent stem cells to investigate cardiac phenotypes in Timothy syndrome. Nature.

[24] Malan D, Friedrichs S (2011). Cardiomyocytes obtained from induced pluripotent stem cells with long-QT syndrome 3 recapitulate typical disease-specific features in vitro. Circ Res.

[25] Sun N, Yazawa M (2012). Patient-specific induced pluripotent stem cells as a model for familial dilated cardiomyopathy. Sci Transl Med.

[26] Lan F, Lee AS (2013). Abnormal calcium handling properties underlie familial hypertrophic cardiomyopathy pathology in patient-specific induced pluripotent stem cells. Cell Stem Cell.

[27] Kim C, Wong J (2013). Studying arrhythmogenic right ventricular dysplasia with patient-specific iPSCs. Nature.

[28] Taura D, Sone M (2009). Induction and isolation of vascular cells from human induced pluripotent stem cells–brief report. Arterioscler Thromb Vasc Biol.

[29] Li Z, Hu S (2011). Functional characterization and expression profiling of human induced pluripotent stem cell- and embryonic stem cell-derived endothelial cells. Stem Cells Dev.

[30] Adams WJ, Zhang Y (2013). Functional vascular endothelium derived from human induced pluripotent stem cells. Stem Cell Reports.

[31] Tan KS, Tamura K (2013). Molecular pathways governing development of vascular endothelial cells from ES/iPS cells. Stem Cell Rev.

[32] Yamamoto T, Shibata R (2013). Therapeutic reendothelialization by induced pluripotent stem cells after vascular injury–brief report. Arterioscler Thromb Vasc Biol.

[33] Kinnear C, Chang WY (2013). Modeling and rescue of the vascular phenotype of Williams-Beuren syndrome in patient induced pluripotent stem cells. Stem Cells Transl Med.

[34] Lieu DK, Fu JD (2013). Mechanism-based facilitated maturation of human pluripotent stem cell-derived cardiomyocytes. Circ Arrhythm Electrophysiol.

